# Improving GPs’ Emotional Intelligence and Resilience to Better Manage Chronic Respiratory Diseases Through an Experiential Online Training Intervention: A Mixed Methods Study

**DOI:** 10.3390/healthcare13010021

**Published:** 2024-12-25

**Authors:** Katerina Boulougari, Antonios Christodoulakis, Izolde Bouloukaki, Evangelos C. Karademas, Christos Lionis, Ioanna Tsiligianni

**Affiliations:** 1 Department of Social Medicine, School of Medicine, University of Crete, 71500 Heraklion, Greece; christodoulakisa@uoc.gr (A.C.); izolthi@gmail.com (I.B.); lionis@uoc.gr (C.L.); i.tsiligianni@uoc.gr (I.T.); 2 Department of Nursing, School of Health Sciences, Hellenic Mediterranean University, 71410 Heraklion, Greece; 3 Department of Psychology, University of Crete, 74150 Rethymno, Greece; karademas@uoc.gr; 4 Department of Social Sciences, University of Nicosia, 1700 Nicosia, Cyprus

**Keywords:** emotional intelligence, motivational interviewing, GPs’ training, soft skills, self-regulation, well-being

## Abstract

Background/Objectives: High levels of emotional intelligence (EI) and resilience in primary care physicians (PCPs) can help them communicate better with patients, build stronger relationships with colleagues, and foster a positive and collaborative workplace. However, studies have indicated that primary care physicians (PCPs) often do not focus enough on developing these skills. Consequently, the purpose of this mixed methods study was to evaluate the effectiveness of an experiential online training (EOT) intervention in enhancing the EI and resilience of PCPs who treat patients with chronic respiratory diseases (CRDs). Methods: A total of 46 PCPs from Greece participated in a 25-hour EOT program, which focused on counseling skills for lifestyle change and breathing techniques for self-regulation. Quantitative data were collected using the Trait Emotional Intelligence Questionnaire-Short Form (TEIQue-SF) and the Connor–Davidson Resilience Scale (CD-RISC-25) before, immediately after, and three months post-intervention. Additionally, qualitative data were obtained through written reflections from participants regarding their clinical practice. Results: The results revealed significant improvements in EI and resilience scores immediately after the intervention (ΕΙ: 5.13, SD: 0.65 vs. 5.3, SD: 0.57, *p* = 0.007; resilience: 76.6, SD: 11.75 vs. 79.83, SD: 10.24, *p* = 0.029), as well as at the three-month follow-up (ΕΙ: 5.3, SD: 0.57 vs. 5.36, SD: 0.48, *p* = 0.007; resilience: 79.83, SD: 10.24 vs. 81.03, SD: 7.86, *p* = 0.029). The thematic analysis of qualitative data identified improvements in five key themes: communication skills, stress management, emotional awareness, resilience, and patient care. Participants reported feeling more confident, empathetic, and effective when interacting with patients, particularly those from diverse backgrounds. The convergence of the quantitative and qualitative findings showed the efficacy of the EOT intervention in enhancing PCPs’ EI, resilience, well-being, and, ultimately, their practice. Conclusions: These findings underscore the importance of integrating EI and resilience training into medical education and professional development programs. Such initiatives could effectively support the emotional and psychological needs of PCPs and potentially contribute to an improved quality of care for patients with CRDs.

## 1. Introduction

According to the World Health Organization (WHO), there has been a rise in the prevalence of chronic respiratory diseases (CRDs) like asthma and chronic obstructive pulmonary disease (COPD) [1,2]. This increase can be attributed to numerous factors such as globalization, urbanization, aging populations, risk factors, and changes in health policies [2,3]. These diseases are particularly worrisome due to their significant impact on the quality of life of patients and mortality and morbidity rates, resulting in over 4 million deaths annually [1,3]. Furthermore, CRDs necessitate substantial resource allocation (i.e., pharmaceutical treatments) to be effectively managed [1,3]. This resource allocation can be reduced through non-pharmacological interventions, with a notable example being having more effective disease control [4,5,6]. The self-control of the disease can be improved through behavioral change and effective communication with physicians [4]. However, there appears to be a lack of communication, as patients have limited knowledge and understanding about their disease [1,4]. Therefore, it is essential to incorporate education into the management of CRDs to empower patients and enable them to take control of their health [7]. To accomplish this, healthcare professionals must shift from a paternalistic role to a patient-centered approach by becoming skilled communicators and effective empathetic trainers and coaches in the healthcare field [7,8]. This transition requires healthcare professionals to possess certain skills, including emotional intelligence and resilience, which are rarely the focus of their clinical education [7,8].

Primary care physicians (PCPs) are the cornerstone of any healthcare system as they are the first point of contact for patients and manage a wide range of health issues, including CRDs [9,10]. However, the demanding nature of their jobs, with a high number of patients with multiple health-related and not health-related problems (social, economic, etc.), limits PCPs’ communication with their patients [11,12,13]. Furthermore, recent criticism has highlighted the shortcomings of primary care, emphasizing the need for focused interventions to improve its effectiveness [14,15]. Therefore, providing PCPs with the necessary skills to cope with the demands of their jobs is crucial for improving the self-control of their patients’ diseases and the quality of the overall healthcare they provide. Unfortunately, the majority of existing interventions primarily focus on knowledge and clinical skill development, patient care, organizational changes, and workload management rather than addressing PCPs’ personal capacity to effectively communicate with their patients and control their disease [16,17,18,19,20]. Thus, interventions that aim to enhance communication skills tend to concentrate on improving one specific skill at a time, and they are usually brief, lasting from a few hours to a few days [21,22]. This short duration makes it difficult to evaluate any changes in abilities, attitudes, and behaviors [21,22]. As a result, there is a lack of interventions that effectively target and improve the crucial communication skills of PCPs, for example, emotional intelligence and resilience, which could make them more effective mentors and coaches.

Emotional intelligence (EI) and resilience are two skills that when adequately cultivated can significantly help PCPs to better communicate with their patients and promote disease control [23,24,25,26,27,28,29]. EI could be defined as “the ability to monitor one’s own and others’ feelings and emotions, to discriminate among them, and to use this information to guide one’s thinking and actions” [30]. On the other hand, resilience is “the process and outcome of successfully adapting to difficult or challenging life experiences, especially through mental, emotional, and behavioral flexibility and adjustment to external and internal demands” [31]. For healthcare professionals, higher levels of EI and resilience can improve patient communication and empathy, enhance interpersonal relationships, and facilitate better conflict resolution [32,33,34,35,36]. However, studies have shown that PCPs do not sufficiently foster these skills, which limits their effectiveness as communicators [37,38].

In Greece, healthcare professionals work in more stressful and demanding clinical environments with various challenges over the years, including economic austerity measures [39,40], than in other countries, which further inhibits their motivation and capacity to properly communicate with their patients and help them self-control their disease [41,42,43,44]. Therefore, and considering the aforementioned, there is an urgent need to develop an intervention that could help PCPs develop their emotional intelligence and resilience, and thus motivate them to evolve as health mentors/coaches, who will ultimately provide higher-quality care to their patients. Towards that end, we have developed an experiential online training (EOT) program, based on Kolb’s learning model [45], for PCPs. This program aimed to improve the emotional intelligence and resilience of PCPs, by empowering them to more effectively communicate, empathize, and better assist patients with CRDs in managing their disease. Consequently, the purpose of this study was to assess the effectiveness of the EOT program in improving PCPs’ emotional intelligence and resilience to better manage patients with CRDs.

## 2. Materials and Methods

### 2.1. Study Design

The current study employed a convergent mixed methods design, which entailed the simultaneous collection of qualitative and quantitative data, followed by separate analysis and joint interpretation [46]. An invitation was extended to Greek medical societies of general practitioners through the participating university. Information about the EOT program and the study was provided to the 49 respondents who expressed interest, and they were subsequently invited to participate. However, 3 participants withdrew from the course due to time constraints and other commitments, resulting in a final sample size of 46. The present 25-hour EOT program, which is described in greater detail in Table 1, had the aim of equipping primary care physicians (PCPs) with practical skills and strategies for managing their emotions, enhancing their communication abilities, and promoting their overall well-being. The program’s duration was from 11 May 2022 to 27 November 2022.

The inclusion criteria for the present study were successful completion of the training by attending at least 80% of the Zoom sessions and willingness to voluntarily participate in the study. On the other hand, the exclusion criteria were the failure to submit a written reflection on practicing motivational interviewing (MI) skills and the non-submission of complete questionnaires. Data were collected at three different time points: before the intervention, immediately after the intervention, and three months following the intervention (follow-up).

### 2.2. Sample Size Calculation

We utilized G*Power (version 3.1) to determine the sample size using a power analysis for a repeated-measures ANOVA with one group and three measurement points (pre-intervention, post-intervention, and three months after the intervention) [47]. We set the parameters to detect a medium effect size (f = 0.25), with an alpha error probability (α) of 0.05 and a power (1 − β) of 0.95. The correlation among repeated measures was set to 0.5. The analysis indicated that a total sample size of 43 participants would be required to achieve the desired effect size. Taking into account an attrition rate of about 15%, we determined that 49 participants were needed. It should be noted that, although 3 participants withdrew, the resulting sample size of 46 remained adequate to achieve the desired effect size.

### 2.3. Data Collection

Participants completed a self-reported questionnaire developed by the authors to collect sociodemographic information. Additionally, the participants provided written reflections on their experiences in clinical settings, offering qualitative insights into the impact of the intervention on their professional practice and personal development. It should be noted that written reflections are commonly utilized as a qualitative data collection method, particularly in educational research [48]. The following validated instruments were also used with permissions obtained from the creators:

#### 2.3.1. Trait Emotional Intelligence Questionnaire-Short Form (TEIQue-SF)

The Trait Emotional Intelligence Questionnaire-Short Form (TEIQue-SF) measures the trait emotional intelligence (EI) [49,50]. It consists of 30 items rated on a 7-point Likert scale (1 = strongly disagree to 7 = strongly agree). The questionnaire assesses four dimensions of EI, i.e., well-being, self-control, emotionality, and sociability, with higher scores indicating higher levels of trait EI. Scores above 5.0 are generally considered indicative of high trait EI, while scores below 3.5 suggest areas for improvement [49,50]. The TEIQue-SF has been translated and validated in Greek [51], and also has been used in previous studies in which it demonstrated good reliability and validity [50,52].

#### 2.3.2. Connor–Davidson Resilience Scale (CD-RISC-25)

The Connor–Davidson Resilience Scale (CD-RISC-25) consists of 25 self-report items and measures resilience [53]. The CD-RISC-25 assesses different aspects of resilience, including control, dedication, self-confidence, perceiving change as a challenge, stable and trustworthy relationships, adaptability to change, developing goal-attainment strategies, appreciating the strength fostered by adversity, recognizing the significance of patience and tolerance for negative emotions, and embracing optimism [53,54]. Each item is rated on a 5-point scale, ranging from 0 (not true at all) to 4 (true nearly all of the time). Scores on the CD-RISC-25 range from 0 to 100, with higher scores reflecting greater resilience. More specifically, scores above 80 are considered indicative of high resilience, while scores below 60 suggest lower resilience [53]. Furthermore, the CD-RISC-25 has been translated and validated in Greek [54].

### 2.4. Statistical Analysis

Descriptive statistics were used to present quantitative variables as mean values with standard deviations (SD), while categorical variables were expressed as absolute and relative frequencies. Repeated measures analysis of variance (ANOVA) was conducted to assess changes in TEIQue-SF and CD-RISC-25 scores over time. Bonferroni correction (*p* = 0.05/3) was used in case of multiple testing to control type I error. All reported *p*-values were two-tailed, and statistical significance was set at *p* < 0.05. The statistical analyses were performed using SPSS statistical software (version 26.0). For the qualitative data, content analysis was employed [55]. This involved coding, sorting, and synthesizing the data from the written reflections to generate themes/categories. The resulting themes/categories were then analyzed by three authors through contrasting and discussing until a consensus was reached [55]. After completing the analyses, both the qualitative and quantitative data were used for triangulation, to examine the convergence or divergence between the two types of data [56]. This process resulted in the creation of a figure depicting the convergence of both data types (quantitative and qualitative).

## 3. Results

### 3.1. Demographics

The majority of the 46 participants were women (65.2%), aged between 45 and 60 years (43.5%), married (65.2%), with children (58.7%), and university alumni (56.5%). Additionally, 45.7% had 10–19 years of working experience, 80.4% were employed in the public sector, and 32.6% had prior experience in soft skills training (Table 2).

### 3.2. Quantitative Findings

**Figure 1:**
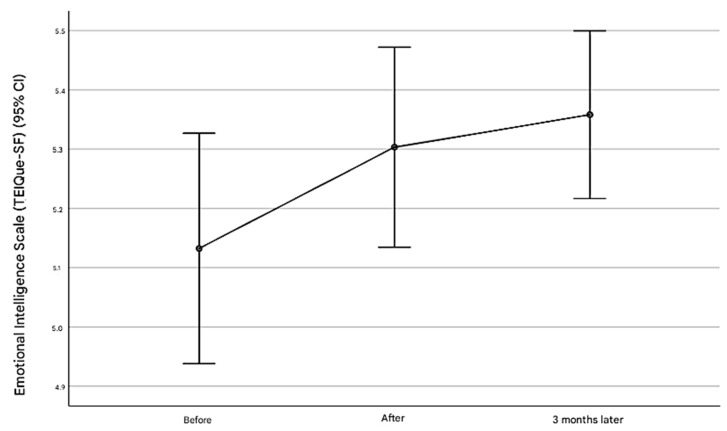
The changes in the emotional intelligence levels of participants pre-intervention, post-intervention, and three months post-intervention.

The results indicated significant improvements in the total EI score, as well as specific dimensions of EI, such as well-being and sociability (Table 2). The mean total EI score increased from 5.13 (SD = 0.65) pre-intervention to 5.30 (SD = 0.57) post-intervention and 5.36 (SD = 0.48) at the three-month follow-up (*p* = 0.007) (Figure 1). Pairwise comparisons indicated significant increases from pre-intervention to post-intervention (*p* = 0.020) and from pre-intervention to three months (*p* = 0.008), with no significant difference between post-intervention and three months (*p* = 0.399). The well-being dimension showed a significant increase from 5.39 (SD = 0.94) pre-intervention to 5.77 (SD = 0.71) post-intervention and 5.70 (SD = 0.59) at the three-month follow-up (*p* = 0.003). Sociability scores also increased significantly from 4.69 (SD = 0.83) pre-intervention to 4.86 (SD = 0.81) post-intervention and 4.92 (SD = 0.88) at three months.

**Figure 2:**
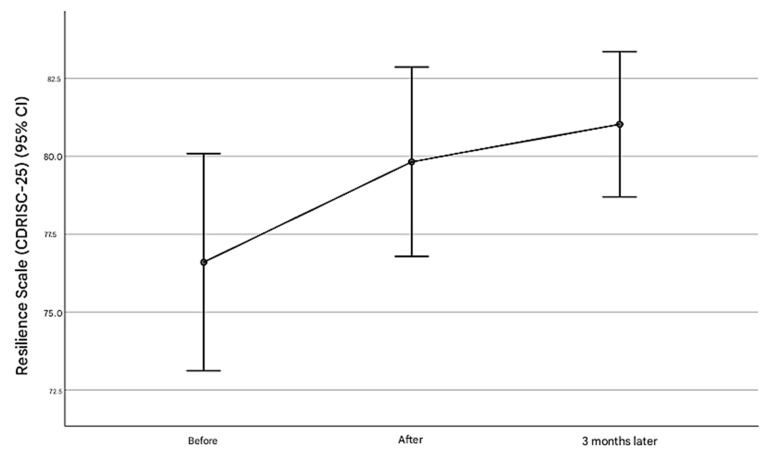
The changes in the resilience levels of participants pre-intervention, post-intervention, and three months post-intervention.

The resilience scores significantly increased over the study period (Table 3). More specifically, the mean resilience score increased from 76.6 (SD = 11.75) pre-intervention to 79.83 (SD = 10.24) post-intervention and 81.03 (SD = 7.86) at the three-month follow-up (Figure 2). Pairwise comparisons revealed significant improvements from pre-intervention to post-intervention (*p* = 0.048) and from pre-intervention to three months (*p* = 0.026), with no significant difference between post-intervention and three months (*p* = 0.412). Furthermore, no significant variations were observed across demographic and professional characteristics of the participants (gender, age, education levels, experience, role/responsibility, sector, area of practice, prior training in soft skills) for either scale, pre-intervention, post-intervention, and three months post-intervention (Supplementary File S1).

### 3.3. Qualitative Findings

The thematic analysis of qualitative data from the participants’ written reflections suggested five themes, which are depicted in Table 4.

#### 3.3.1. Theme 1: Enhanced Communication Skills

Participants reported improved communication skills with patients, feeling more confident when using motivational interviewing techniques, and empathizing with patients. For example, one participant noted, “*I saw an improvement in my communication skills. I learned to listen more; I improved in open questions. I feel relieved when the patient chooses the agenda and the burden of having to direct him goes away*” (KG group 2).

#### 3.3.2. Theme 2: Improved Stress Management

The self-regulation techniques, particularly the breathing exercises taught during the intervention, were highlighted as effective tools for managing stress. A participant reflected, “*The breathing exercises have been a game-changer for me. They help me stay calm and focused, even during the most stressful consultations*” (AN group 2).

#### 3.3.3. Theme 3: Increased Emotional Awareness

Participants reported greater awareness of their own emotions and how these emotions affected their interactions with patients. One participant stated, “*…I also noticed that the whole process of discussion with the patient is clearly less stressful for me, while my support in framing the discussion reduces my stress and self-criticism about my levels of effectiveness*” (TN group 3).

#### 3.3.4. Theme 4: Greater Resilience

The intervention positively impacted participants’ resilience, making them better able to cope with work demands. A participant shared, “*The speed of the conversation of both myself and the patient suddenly changed and also our mood, as soon as I applied the breathing techniques for self-regulation*” (VI group 3).

#### 3.3.5. Theme 5: Positive Impact on Patient Care

Improvements in EI and resilience had a direct positive impact on patient care. Participants reported being more patient-centered and effective in their consultations. For example, one participant remarked, “…*I believe this conversation was more meaningful than our previous discussions on the same topic, even though once again, the patient did not commit to the ultimate goal of quitting smoking*” (ΤΧ group 1).

### 3.4. Triangulation of Quantitative and Qualitative Data

**Figure 3:**
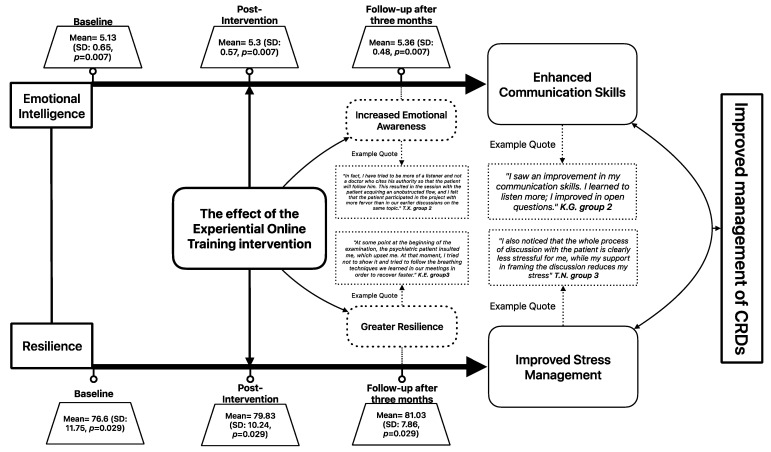
Triangulation of quantitative and qualitative data.

Figure 3 illustrates how the integration of quantitative and qualitative findings contributes to a comprehensive understanding of the impact of the EOT intervention. The improvement in EI and resilience scores was supported by qualitative data, indicating enhanced communication skills, improved stress management, increased emotional awareness, and greater resilience among participants. When combined with the quantitative data, these findings strongly suggest that our EOT intervention effectively achieved its objectives and had a significant and sustainable effect (for at least 3 months) on the professional practice and personal well-being of the participants (Figure 3).

## 4. Discussion

The present study aimed to assess the effectiveness of an EOT program in improving EI and resilience among PCPs to better manage patients with CRDs. Our findings indicate that there were significant improvements in both EI and resilience scores immediately after the intervention and at the three-month follow-up. More specifically, the quantitative data suggested a significant increase in PCPs’ emotional intelligence, leading to greater empathy and improved communication with patients, potentially resulting in better patient outcomes. Additionally, there was a noticeable increase in resilience scores which was reinforced by the qualitative data, suggesting that PCPs felt better equipped to manage the stresses, and potentially being more effective in communicating. Moreover, the qualitative data from written reflections further suggested that our program contributed to better patient interactions and improved the communication skills of PCPs. Therefore, this study contributes to the existing literature by highlighting the importance of improving the EI and resilience of PCPs and emphasizing the need for targeted interventions to support the emotional and psychological needs of PCPs to better manage patients with CRDs.

A major finding of the present study was that EI could be improved through an EOT intervention in PCPs and, in turn, improved how they managed their patients’ CRDs. The qualitative data further suggested that our interventions improved the communication skills of PCPs. This finding aligns with the current literature that emphasizes the importance of improving EI in healthcare professionals, since higher EI is positively associated with caring behaviors and better communication [35,57]. Moreover, previous studies have shown that higher levels of EI were associated with improved clinical performance and better patient relationships [36,58]. Another study suggested that healthcare professionals with higher EI scores have higher self-esteem, which in turn improved their well-being and health [59]. Similarly, another study highlighted that EI improves the healthcare professionals’ ability to provide safe and empathetic healthcare, which is increasingly crucial in today’s healthcare landscape [60]. Our study further supports these findings by demonstrating that the EOT intervention effectively enhanced EI, leading to improved patient management, interactions, and communication skills. A possible explanation for this finding could be that improved communication skills, such as empathy, fostered a better understanding and trust between physicians and patients, enhancing patient management and overall healthcare experience [61].

Another major finding of the present study was the increase in resilience among participants. This finding is also consistent with previous research on the role of resilience in healthcare professionals [60,62,63,64]. For example, a study found that resilience was negatively associated with burnout [62]. Similarly, our study showed that primary care physicians felt more capable of coping with the stresses and challenges of their profession after the EOT intervention. This improvement in resilience could be attributed to the incorporation of breathing techniques for self-regulation, as suggested by our qualitative findings, which many participants noted to have helped them be calm and focused during stressful situations of everyday clinical practice. This finding is further supported by other studies, which suggested that resilience and stress management programs that incorporate breathing techniques significantly reduce blood pressure and improve the emotional well-being of participants [63,64]. Furthermore, participants reported that the improvements in their EI and resilience had a direct positive impact on their communication with their patients, making them more patient-centered, empathetic, and effective in their consultations. One possible explanation for this finding could be that, in order to achieve successful non-pharmaceutical treatment, PCPs need to adopt a more empathetic approach rather than the usual impersonal one [65]. This empathetic approach could then help facilitate successful behavioral change in patients with COPD, resulting in improvements in the quality of life for these patients [65]. Interestingly, another study has suggested that an intervention could improve EI and decrease stress in healthcare professionals and, in turn, enhance the quality of life for their patients [60].

Our experiential online intervention, based on our findings, was successful in improving the EI and resilience of PCPs. This could be attributed to the flexibility and accessibility of online formats, which make them an attractive option for busy healthcare professionals, thus enabling them to attend training without disrupting their clinical duties [66]. On the other hand, the sustained improvements in EI and resilience observed at the three-month follow-up were consistent with findings from resilience training in other similar training programs [67]. This long-term impact suggests that the skills and techniques learned during the intervention were effectively integrated into the participants’ daily practices. A potential explanation for this finding could be that the ongoing practice and reinforcement of EI and resilience techniques helped embed these skills more deeply, leading to lasting behavioral changes (at least three months after the intervention) [66].

Policymakers and healthcare managers could utilize our findings and incorporate EI and resilience training into medical education and continuing professional development programs. Therefore, providing ongoing professional development opportunities, particularly through flexible and accessible online platforms [66], could help PCPs continually improve their skills without disrupting their schedules and clinical responsibilities. Moreover, promoting self-regulation techniques, such as breathing exercises, could assist PCPs in managing stress in real time, resulting in reduced burnout and increased job satisfaction [68]. In terms of future research, it would be beneficial to explore the long-term effects of EI and resilience training on PCPs’ well-being and improvement in CRD care outcomes. Therefore, multi-centered cohort studies could cement the use of similar EOT interventions in PCPs. Additionally, studying the effectiveness of different training formats and content, as well as examining specific patient outcomes, such as adherence to lifestyle changes and patient with CRD satisfaction with their care, could provide valuable insights into the broader benefits of these interventions. Consequently, further research could focus on developing more specific and efficient approaches to promote the well-being of PCPs and improve their communications skills for patients with CRDs.

### Limitations

Our study provides an EOT program (intervention) that could improve the emotional intelligence and resilience of PCPs, leading to improved management for patients with CRDs. Moreover, to the best of our knowledge, this is the first EOT intervention study taking place in Greece, with the aim of improving the EI and resilience of PCPs to better manage patients with CRDs. Nevertheless, and despite its strengths, our study has a few limitations. First, the sample size was relatively small and limited to PCPs from Greece, which may limit the generalizability of the findings to other contexts and healthcare systems. Second, our study followed PCPs only after three months, and therefore, we cannot safely say about the sustainability of our intervention after the three-month mark. Third, the study’s reliance on self-reported measures may also have introduced self-report bias into both the data and its analysis. Fourth, the online format could be a barrier when implementing this intervention to PCPs who are not adequately versed in the use of computers, although this should be relatively rare. Fifth, our results may have been affected by participant personality biases, for instance, introversion or extroversion. Finally, since participants were volunteers, they may have been highly motivated to learn and adopt the new knowledge and skills of the intervention, which may have had a positive impact on the effectiveness of the intervention. Consequently, future studies could benefit from larger sample sizes, diverse populations, and the use of objective measures of EI and resilience, such as performance-based assessments.

## 5. Conclusions

In conclusion, this study presents an experiential online training intervention program that, according to our findings, has the potential to enhance the emotional intelligence and resilience of primary care practitioners. This improvement in their abilities could enable them to better communicate with patients who have chronic respiratory diseases. Additionally, our results emphasize the potential of incorporating emotional intelligence and resilience training into medical education and professional development programs. They also highlight the ongoing need for support and education for healthcare providers. This is crucial because enhancing the emotional intelligence and resilience of primary care practitioners could enable them to better comprehend and address the complex requirements of their patients, resulting in more effective and compassionate care. Consequently, healthcare managers could utilize our experiential online training program and findings to assist primary care practitioners in enhancing these fundamental communication skills and, ultimately, the quality of care they would provide to patients with chronic respiratory diseases.

## Figures and Tables

**Table 1:** Brief descriptions and expected outcomes of each module of the EOT intervention utilized in this study.

Module	Short Description of the Module	Methods/Tools	Expected Outcomes
Baseline Needs Assessment	Conduct a Training Needs Assessment (TNA) to evaluate primary care practitioners’ (PCPs) current competencies in managing chronic respiratory diseases and ascertain their educational requirements	Utilize the questionnaires of the study	Establish baseline knowledge, skills, and areas of improvement
Module 1: Empathy and Resilience in Therapeutic Communication	Provide training for PCPs in emotional intelligence (EI) and patient-centered communication strategies for the management of chronic respiratory diseases such as COPD and asthma	Interactive sessions focusing on empathy, reflective practice, and patient engagement utilizing Zoom and Moodle platforms	Enhance EI skills and patient-centered care approach
Module 2: Building Trust and Motivational Interviewing (MI)	Introduce/teach MI principles, emphasizing behavior modification and establishing rapport with patients	Sessions include role playing, simulations, and group discussions on MI	Enhance communication skills and increase patient motivation for behavioral modification
Module 3: Communication Tools in MI and Brief Intervention	Provide tools and strategies for collaborative decision making and brief interventions	Practical exercises on therapeutic action planning and developing co-created behavior change pathways	Empower patients, enhance therapeutic outcomes, and promote shared decision making
Module 4: Resilience and Stress Management Techniques	Improve resilience through self-regulation, mindfulness, and stress management techniques, particularly using breathing technique	Mindfulness sessions, self-regulation exercises, and discussions on emotional resilience in managing COPD/asthma	Help PCPs manage stress and improve their emotional well-being and patient care
Module 5: Practical Application	PCPs implement the acquired competencies in clinical settings, interacting with patients to promote behavioral modifications	Direct patient interaction and practical application of MI and self-regulation techniques	Enhance practical competencies in behavior change communication and resilience techniques
Module 6: Presentation and Reflection	Participants present their experiences with practical applications in focus groups and reflect on the intervention’s efficacy	10-minute presentations by PCPs followed by group discussions and peer feedback	Reinforce learning, promote knowledge sharing, and encourage continuous improvement
Module 7: Evaluation and Monitoring	Assess the program’s efficacy through post-intervention evaluations (immediately upon program completion and after a three-month interval)	Utilization of evaluation questionnaires, reflective documentation, and a knowledge assessment of MI	Evaluate learning outcomes, ensure objectives are achieved, and provide feedback for program enhancement
Certification and Credit	Award participants a certificate of successful completion and European credit system for vocational education and training (ECVET) points	Formal recognition of program completion and educational credits	Provide motivation and professional development recognition

**Table 2:** Demographic characteristics of the participants.

		*n*	%
Sex	Female	30	65.2
	Male	16	34.8
Age	25–30	9	19.6
	31–44	17	37.0
	45–60	20	43.5
Level of education	University degree	26	56.5
	Master	17	37.0
	Ph.D.	3	6.5
Marital status	Single	14	30.4
	Married	30	65.2
	Divorced	1	2.2
	Widower	1	2.2
Do you have children?	No	19	41.3
	Yes	27	58.7
Work experience (years)	1–9	18	39.1
	10–19	21	45.7
	20–29	6	13.0
	30+	1	2.2
Do you specialize in chronic respiratory diseases?	No	44	95.7
	Yes	2	4.3
Have you trained in soft skills before?	No	31	67.4
	Yes	15	32.6

**Table 3:** Changes in participants’ emotional intelligence and resilience pre-intervention, post-intervention, and 3 months after the intervention.

	Pre	Post	3 Months	*p* *	*p* ** Pre vs. Post	*p* ** Pre vs. 3 Months	*p* ** Post vs. 3 Months
	Mean (SD)	Mean (SD)	Mean (SD)				
***Total TEIQue-SF score***	5.13 (0.65)	5.30 (0.57)	5.36 (0.48)	0.007	0.020	0.008	0.399
Emotionally	5.21 (0.79)	5.28 (0.7)	5.36 (0.54)	0.299	-	-	-
Self-control	4.87 (0.97)	4.93 (0.78)	5.05 (0.72)	0.223	-	-	-
Well-being	5.39 (0.94)	5.77 (0.71)	5.7 (0.59)	0.003	0.001	0.020	0.349
Sociability	4.69 (0.83)	4.86 (0.81)	4.92 (0.88)	0.049	0.076	0.041	0.521
***Total CDRISC-25 score***	76.6 (11.75)	79.83 (10.24)	81.03 (7.86)	0.029	0.048	0.026	0.412

* *p*-value from repeated measures ANOVA; ** *p*-value for pairwise comparisons after Bonferroni correction. *p* = 0.05.

**Table 4:** The five themes of our study and example quotes.

Theme	Example Quote
Enhanced Communication Skills	“*In fact, I have tried to be more of a listener and not a doctor who cites his authority so that the patient will follow him. This resulted in the session with the patient acquiring an unobstructed flow, and I felt that the patient participated in the project with more fervor than in our earlier discussions on the same topic…*” (TX group 2)
	“*…I saw an improvement in my communication skills. I learned to listen more; I improved in open questions. I feel relieved when the patient chooses the agenda and the burden of having to direct him goes away.*” (KG group 2)
Improved Stress Management	“*I think that despite its difficulties and failures on my part, our discussion, gave the patient, the opportunity to express the values that are important to him.*” (SP group 1)
	“*…I also noticed that the whole process of discussion with the patient is clearly less stressful for me, while my support in framing the discussion reduces my stress and self-criticism about my levels of effectiveness.*” (TN group 3)
Increased Emotional Awareness	“*…I still think I need to become less directive and more supportive because I often fall into the trap of talking more and as a result, I sometimes become critical. Eventually I realized that what I think is optimal for my patient is not always what they need.*” (PI group 2)
	“*…This concern, that I genuinely expressed about his cough, especially with someone I was seeing for the first time, laid the foundation for building a trusting relationship that can yield significant results. It is certain that more meetings with this patient will be needed, but I am hopeful that after our first communication the way has been paved to attempt a behavior change intervention to try to stop smoking.*” (AI group 3)
Greater Resilience	“*…At some point at the beginning of the examination, the psychiatric patient insulted me, which upset me. At that moment, I tried not to show it and tried to follow the breathing techniques we learned in our meetings in order to recover faster. I believe that the fact that the patient did not understand my discomfort, contributed positively to our work together. In the future I will aim to practice my resilience, as I find that communicating with a demanding or abusive patient affects my mood.*” (KE group 3)
	“*…The speed of the conversation of both myself and the patient suddenly changed and also our mood, as soon as I applied the breathing techniques for self-regulation.*” (VI group 3)
Positive Impact on Patient Care	“*…When I saw the satisfaction on the patient’s face as he said goodbye, it was a significant moment for my own sense of empowerment. The feeling of confidence that everything would improve with his asthma. The fact that he had shared something that had clearly been worrying him, but he had been afraid to discuss before. All of this became a source of energy for me to continue giving my best as a doctor.*” (AΜ group 1)
	“*I consciously chose a ’difficult’ patient with whom I have repeatedly discussed quitting smoking. This time, I intentionally spoke less than the patient. I believe this conversation was more meaningful than our previous discussions on the same topic, even though once again, the patient did not commit to the ultimate goal of quitting smoking.*” (ΤΧ group 1)

## Data Availability

The data that support the findings of this study are available from the corresponding author upon reasonable request.

## Supplementary Materials

The following supporting information can be downloaded at: https://www.mdpi.com/article/10.3390/healthcare13010021/s1, Table S1: Changes in participants’ emotional intelligence across demographic and professional characteristics of the participants, pre, post, and 3 months after the intervention; Table S2: Changes in participants’ resilience across demographic and professional characteristics of the participants, pre, post, and 3 months after the intervention.
