# Supplementary material for: Improving GPs’ Emotional Intelligence and Resilience to Better Manage Chronic Respiratory Diseases Through an Experiential Online Training Intervention: A Mixed Methods Study
Source: Healthcare (Basel). 2024 Dec 25;13(1):21. doi: 10.3390/healthcare13010021 (PMC11720043; doi:10.3390/healthcare13010021)
Supplement: Supplementary file 1 [file healthcare-13-00021-s001.zip › healthcare-3356478-supplementary.pdf]

Table S1. Changes in participants' emotional intelligence across demographic and professional characteristics of the participants, pre, post, and 3 months after the intervention.

Variable	Before - Mean (SD)	After - Mean (SD)	3 Months Later - Mean (SD)	p-value[1]	p-value[2]
**Gender**					
Female	5.07 (0.66)	5.21 (0.62)	5.31 (0.56)	0.077	0.719
Male	5.25 (0.66)	5.47 (0.42)	5.45 (0.24)	0.189	
**Age**					
25–44 years	5.32 (0.43)	5.37 (0.51)	5.43 (0.44)	0.607	0.099
45–60 years	4.89 (0.81)	5.21 (0.64)	5.27 (0.51)	0.005	
**Educational Level**					
University Degree	4.99 (0.64)	5.23 (0.57)	5.32 (0.36)	0.012	0.268
Master's/Doctoral Degree	5.31 (0.64)	5.40 (0.56)	5.41 (0.60)	0.701	
**Years of Work Experience**					
1–9 years	5.05 (0.70)	5.19 (0.58)	5.37 (0.36)	0.060	0.389
10+ years	5.18 (0.63)	5.37 (0.56)	5.35 (0.54)	0.130	
**Role Responsibility**					
Resident	5.17 (0.55)	5.13 (0.63)	5.30 (0.34)	0.359	0.264
Attending/Coordinator/Director	4.98 (0.73)	5.29 (0.56)	5.30 (0.55)	0.006	
Private Practitioner	5.59 (0.32)	5.71 (0.25)	5.67 (0.35)	0.767	
**Public Sector Employment**					
No	5.59 (0.29)	5.71 (0.23)	5.63 (0.34)	0.733	0.492
Yes	5.04 (0.67)	5.22 (0.58)	5.30 (0.48)	0.018	
**Area of Practice**					
Semi-urban/Rural	5.17 (0.56)	5.38 (0.45)	5.40 (0.51)	0.157	0.902
Urban	5.11 (0.72)	5.25 (0.64)	5.33 (0.46)	0.123	
**Prior Soft Skills Training**					
No	5.09 (0.72)	5.34 (0.62)	5.39 (0.51)	0.006	0.162
Yes	5.23 (0.49)	5.22 (0.44)	5.28 (0.41)	0.866	

[1]p-value for comparisons between time measurements after Bonferroni correction. [2]p-value from repeated ANOVA measurements. Differences in variation from one metric to another between groups.

Table S2. Changes in participants' resilience across demographic and professional characteristics of the participants, pre, post, and 3 months after the intervention.

Variable	Before - Mean (SD)	After - Mean (SD)	3 Months Later - Mean (SD)	p-value[1]	p-value[2]
**Gender**					
Female	75.63 (11.16)	79.27 (10.95)	81.08 (8.75)	0.088	0.717
Male	78.41 (12.94)	80.86 (9.02)	80.95 (6.10)	0.674	
**Age**					
25–44 years	76.64 (11.18)	78.97 (10.18)	80.70 (8.98)	0.307	0.836
45–60 years	76.55 (12.74)	80.93 (10.49)	81.46 (6.31)	0.197	
**Educational Level**					
University Degree	74.91 (13.41)	77.74 (10.55)	80.59 (7.03)	0.098	0.517
Master's/Doctoral Degree	78.80 (9.01)	82.53 (9.41)	81.61 (8.98)	0.356	
**Years of Work Experience**					
1–9 years	73.33 (14.47)	74.98 (10.77)	81.24 (7.49)	0.011	0.048
10+ years	78.70 (9.30)	82.94 (8.72)	80.90 (8.23)	0.122	
**Role Responsibility**					
Resident	73.64 (12.41)	74.20 (11.24)	78.80 (7.65)	0.203	0.721
Attending/Coordinator/Director	76.96 (12.38)	81.69 (8.97)	81.49 (7.36)	0.125	
Private Practitioner	81.23 (6.47)	84.40 (8.86)	83.86 (9.91)	0.766	
**Public Sector Employment**					
No	82.58 (7.10)	85.23 (8.53)	83.51 (9.22)	0.766	0.600
Yes	75.34 (12.20)	78.69 (10.31)	80.51 (7.58)	0.064	
**Area of Practice**					
Semi-urban/Rural	76.61 (11.73)	80.42 (9.50)	79.79 (8.49)	0.380	0.677
Urban	76.59 (11.97)	79.45 (10.85)	81.83 (7.48)	0.124	
**Prior Soft Skills Training**					
No	76.13 (13.18)	81.46 (10.26)	81.85 (8.33)	0.027	0.196
Yes	77.57 (8.35)	76.44 (9.66)	79.34 (6.74)	0.535	

[1]p-value for comparisons between time measurements after Bonferroni correction. [2]p-value from repeated ANOVA measurements. Differences in variation from one metric to another between groups.
